# Nitrate Leaching Mitigation Options in Two Dairy Pastoral Soils and Climatic Conditions in New Zealand

**DOI:** 10.3390/plants11182430

**Published:** 2022-09-17

**Authors:** Dumsane Themba Matse, Paramsothy Jeyakumar, Peter Bishop, Christopher W. N. Anderson

**Affiliations:** Environmental Sciences Group, School of Agriculture and Environment, Massey University, Private Bag 11 222, Palmerston North 4442, New Zealand

**Keywords:** organic inhibitors, gibberellic acid, urine patches, nitrate leaching

## Abstract

This lysimeter study investigated the effect of late-autumn application of dicyandiamide (DCD), co-poly acrylic-maleic acid (PA-MA), calcium lignosulphonate (LS), a split-application of calcium lignosulphonate (2LS), and a combination of gibberellic acid (GA) and LS (GA + LS) to reduce N leaching losses during May 2020 to December 2020 in lysimeter field sites in Manawatu (Orthic Pumice soil) and Canterbury (Pallic Orthic Brown soil), New Zealand. In a second application, urine-only, GA only and GA + LS treatments were applied during July 2020 in mid-winter on both sites. Results showed that late-autumn application of DCD, 2LS and GA + LS reduced mineral N leaching by 8%, 16%, and 35% in the Manawatu site and by 34%, 11%, and 35% in the Canterbury site, respectively when compared to urine-only. There was no significant increase in cumulative herbage N uptake and yield between urine-treated lysimeters in both sites. Mid-winter application of GA and GA + LS reduced mineral N leaching by 23% and 20%, respectively in the Manawatu site relative to urine-only treated lysimeters, but no significant reduction was observed in the Canterbury site. Our results demonstrated the potential application of these treatments in different soils under different climate and management conditions.

## 1. Introduction

Nitrogen (N) leaching from agricultural systems is a global environmental concern. In New Zealand, pastoral dairy farming is mainly characterised by dairy cows feeding outside all year round in pastures mainly dominated by perennial ryegrass (*Lolium perenne* L.) and white clover (*Trifolium repens* L.) [1]. However, dairy cows only utilise small quantities (5–30%) of ingested N from these pastures and a higher proportion (70–95%) of N is excreted in their urine resulting in small areas of highly concentrated N in pastures known as urine patches [2]. The urinary N concentration at each urine patch ranges from 200 to 2000 kg N ha ^−1^ [3] and usually this N rate exceeds plant N uptake. Therefore, the residual N becomes susceptible to leaching as nitrate (NO_3_^−^ -N) into water sources. Nitrate concentrations greater than 11.2 mg NO_3_^−^ -N L^−1^ in both surface and drinking water are deemed harmful to both human and animal health [4]. While concentrations above 0.4 mg L^−1^ NO_3_^−^ -N can also accelerate algal blooms and eutrophication of water bodies [5], thus reducing water quality. Decreasing the amount of NO_3_^−^ -N leaching from urine patches is therefore important for lowering the environmental impact.

Different approaches have been developed and implemented to minimise N losses from grazed pastures [6,7]. The use of nitrification inhibitors, such as dicyandiamide (DCD, C_2_H_4_N_4_) and 3,4-dimethypyrazole phosphate (DMPP, C_5_H_11_N_2_O_4_ P) have been shown to reduce urine patch NO_3_^−^ -N leaching in soils that have a high risk of leaching [7,8]. Reductions ranging from 10% to 76% relative to untreated urine patches have been shown in lysimeter studies. In literature, nitrification inhibitors have been shown to reduce NO_3_^−^ -N leaching through reducing the first step of the nitrification process: the oxidation of ammonia (NH_4_^+^) to hydroxylamine (NH_2_OH). However, in practice, a range of regulatory and technical constraints have limited the widespread use of nitrification inhibitors. Companies voluntarily withdrew sales of DCD in New Zealand following the detection of DCD residues in export milk powder in 2012 [9]. While, for DMPP, efficiency is known to be highly influenced by site conditions such as soil property and climate which implies that widespread deployment is difficult [10,11]. Therefore, there is still a need to develop new inhibitors to reduce the environmental consequences associated with dairy farming.

In a recent incubation study [12] we found that a group of organic compounds have the ability to inhibit nitrification. Our results demonstrated that application of calcium lignosulphonate (LS, C_20_H_24_CaO_10_S_2_) and co-poly acrylic-maleic acid (PA-MA, C_9_H_14_O_6_) can slow nitrification by reducing bio-available Cu. Calcium lignosulphonate is derived from the wood pulp industry and contains high levels of phenolic groups, while PA-MA is an acrylic acid-maleic acid copolymer solution. These compounds have shown a great potential to inhibit nitrification in a controlled environment and reduce the potential for leaching. Hence, this is the first field study conducted to evaluate the effectiveness of LS and PA-MA in reducing NO_3_^−^ -N leaching from urine patches under a wide range of soils and climatic conditions.

However, the main challenge in reducing NO_3_^−^ -N leaching in New Zealand is that the peak NO_3_^−^ -N leaching period in grazed pastural systems is during periods where pasture N uptake is slow due to the low temperatures (winter season). In order to overcome such a shortfall in N uptake, Parsons et al. [13] proposed that application of a plant growth stimulant, Gibberellic acid (GA,C_7_H_6_O_5_), that could help enhance plant growth and subsequent pasture N uptake. However, only Woods et al. [14] examined the potential effect of GA in reducing N leaching. This study found that GA application to Italian ryegrass did not significantly reduce the amount of total NO_3_^−^ -N leaching. This suggests that GA alone is not an effective treatment in reducing N leaching; an additional inhibitor might need to be applied with GA.

To address the recognised research gaps, the current study was conducted to determine the potential effect of nitrification inhibitors and to increase plant growth on NO_3_^−^ -N leaching from dairy cow urine patches in different soils, environment, and management conditions. We hypothesise that (1) application of nitrification inhibitors might reduce nitrification in the soil, thus decreasing NO_3_^−^ -N leaching and (2) the application of GA will reduce the excess of NO_3_^−^ -N by increasing N utilisation by pasture during periods of low N uptake and thus limiting N leaching.

## 2. Materials and Methods

### 2.1. Experimental Sites and Soils

This field lysimeter research was conducted at two different geographic locations: Massey University, Palmerston North, Manawatu (40°23′0.95″ S 175°36′36.16″ E) in the North Island, and Hororata, Canterbury (43°34′13.15″ S, 171°55′47.33″ E) in the South Island of New Zealand (Figure S1). The Manawatu lysimeters soil columns contained intact Orthic Pumice soil [15] collected from Wairakei, and transported to the Manawatu lysimeter facility. The Orthic Pumice soil has low bulk density and is well-drained with high plant available water holding capacity (150–200 mm). The Canterbury lysimeters soil columns were intact Lismore Stony silt loam (Pallic Orthic Brown soil) [15] collected from Hororata. This soil is characterised by an average bulk density and low plant available water holding capacity (40–50 mm) and consists of a shallow layer of fine soil at the top surface, below which the gravel content layer increases significantly. This profile makes the Pallic Orthic Brown soil free draining. The soils selected for this study are representative of soils supporting the highest dairy cow numbers in New Zealand: stock at Canterbury and Waikato dairy farms represent 19.7% and 22.4% of total dairy cows in New Zealand, respectively [16]. In addition, these soils present different properties in terms of water holding capacities which can influence the rate of leaching.

### 2.2. Lysimeters Collection and Pastures

The research described in this paper was undertaken in a lysimeter facility established in May 2019 at both locations. The lysimeter facility at each experimental site consisted of forty-four (44) undisturbed monolith lysimeters made from polyvinyl chloride (PVC) tubes with an internal diameter of 500 mm and depth of 600 mm. Monolith soil columns at both locations were collected following the procedure outlined by Di et al. [17] and installed in a trench facility. A soft wax coat was used between the walls of the PVC casing and the soil to prevent edge flow effects [18].

The pasture at the Manawatu lysimeter facility was Italian ryegrass and at the Canterbury lysimeter facility it was perennial ryegrass and white clover. These two pasture compositions followed farmer practice in the respective area. The soil in each lysimeter was analysed for soil fertility parameters before the application of treatments (Table 1). Based on the initial soil fertility results, the Manawatu site Orthic Pumice soil was low in magnesium (Mg), thus 10 g of Nitrophoska fertilizer (12:5.2:14 + S; Mg; and trace elements) was applied to each lysimeter at the Manawatu facility only on 06 March 2020

### 2.3. Experimental Design

At each experimental site, two sets of experiments were conducted at two different seasonal periods (late-autumn and mid-winter). The late-autumn treatment used twenty-eight (28) lysimeters at each experimental site with the aim to reduce NO_3_^−^ -N leaching during the wet and cold periods of the year (i.e., autumn-winter-spring) [17]. The mid-winter treatment application used sixteen (16) lysimeters at each site and it was aimed to test the effectiveness of GA and its combination with LS on growth during the winter period. Previous studies have indicated that GA can perform better in increasing yield when applied in winter temperatures [19]. The experimental design was a completely randomised block design.

### 2.4. Treatments Application

To simulate urine application by dairy cows, synthetic urine was prepared by dissolving urea (11 g L^−1^), glycine (2.90 g L^−1^), KHCO_3_ (13.98 g L^−1^), KCl (5.04 g L^−1^), and K_2_SO_4_ (1.38 g L^−1^) in water [20] producing a final N concentration of 6 g N L^−1^. Prior to urine application in each period, the grass was cut to 5 cm above the soil surface and lysimeter leachate was collected in both experimental sites to determine leachate NO_3_^−^ -N concentration, to ensure there was no background N (Tables S1 and S2). Urine was applied at a rate of 2 L per lysimeter (equivalent to 10 L m^−2^ or 600 kg N ha^−1^). Control lysimeters received an equal volume of water (2 L).

#### 2.4.1. Experiment 1-Late-Autumn Treatments Application

The first treatment application (late-autumn) was made on 9 June 2020 for the Manawatu site, and 27 May 2020 for the Canterbury site. Seven treatments outlined in Table 2 were applied at each experimental location. In this study, DCD was used as a reference material in terms of reducing NO_3_^−^ -N leaching. At both experimental sites, treatments were applied as a surface spray to each designated lysimeter, 4 h following the urine application. In all lysimeters, 5 mm of water was applied after treatment application to wash applied treatments from pasture canopy and to help distribute treatments in the soil [21].

#### 2.4.2. Experiment 2-Mid-Winter Treatments Application

The second treatment application (mid-winter with air temperature less than 10 °C) was on 29 July 2020 for the Manawatu site and on 26 August 2020 for the Canterbury site. The treatments as outlined in Table 3 were applied as discussed in Section 2.4.1.

### 2.5. Drainage Water Collection and Analysis

Drainage water from each lysimeter was collected into 20 L black plastic containers connected to the base of each lysimeter via a drainage pipe. Drainage water was collected after each heavy rainfall event (>20 mm). The drainage water volume was measured and a sub-sample of approximately 30 mL was collected, filtered, and stored at <4 °C prior to analyses. All samples were analysed within one week of collection for mineral N (NH_4_^+^ and NO_3_^−^) using an Technicon autoanalyzer [22].

### 2.6. Dry matter (DM) Yield and N Uptake Analysis

The timing of herbage harvest from lysimeters was based on regional grazing practice. This resulted in five harvests at Manawatu and four at Canterbury for the late-autumn treatments. For mid-winter treatments there were four harvests from Manawatu and three from Canterbury. During harvest, herbage was cut to a height of 5 cm and the dry weight was recorded after samples were oven-dried at 65 °C for a week.

Oven-dried herbage was homogenised using a Foss^TM^ Cyclotech mill (Thermo Fisher Scientific, Waltham, MA, USA) and passed through a 1 mm sieve. A sub-sample of ground biomass (0.1 g) was analysed for N concentration using the Kjeldahl N method [23].

### 2.7. Soil Mineral N

At the end of the experimental period six soil cores (0–60 cm) were collected from each lysimeter using a stainless-steel corer with internal diameter of 3 cm. The soil cores from each lysimeter were combined to form a composite sample. The composite samples were mixed manually and then sieved through a 2 mm sieve before a 5 g sub-sample was taken for mineral N analysis. Soil samples were extracted using 30 mL, 2 M KCl on an end-over shaker for 1 h. The tubes were then centrifuged at 1100 g for 10 min and filtered through Whatman 42 filter paper. Samples were analysed for mineral N (NH_4_^+^ and NO_3_^−^) using a Technicon autoanalyzer [22]. The autoanalyzer used two colorimetric methods: NH_4_^+^ -N was determined using an indophenol method based on the reaction of NH_3_ with hypochlorite and phenol/salicylate catalysed by nitroprusside. Nitrate was determined using the reduction of nitrate to nitrite by hydrazine followed by the reaction of nitrite with N-(1-naphthyl)-ethylenediamine dihydrochloride to form an azo dye. The resulting colors produced were measured using individual colorimeters [24] and voltage outputs were converted to concentration using a computerized data aquations system (USB-1208FS analog to digital converter and DAQami™ software, Measurement Computing Corporation, Norton, MA, USA) [24].

### 2.8. Climatic Data

Climatic data for the experimental period at both sites were downloaded from the National Institute of Water and Atmosphere (NIWA) database (cliflo.niwa.co.nz) and rainfall was measured onsite using installed rain gauges.

### 2.9. Statistical Analysis

Statistical analyses were done using Minitab (Version 19. Minitab Inc., State College, PA, USA). The treatment comparison effects were analysed using an ANOVA and significant (*p* < 0.05) differences between means were determined using Tukey’s post-hoc test. The percentage of N recovered from applied urine N during in this study was calculated using the following equation [25]:

(1) $$\%\;N\;\mathrm{recovered}\;\mathrm{from}\;\mathrm{applied}\;\mathrm{urine}\;\;N\;=\frac{N_{UR}-N_{0}}{N_{U}}\;\times 100$$

where N_UR_ and N_0_ represents cumulative N output (leached N, soil residual N, and herbage N uptake) in urine-treated lysimeters and control, respectively and N_U_ represents applied urine N concentration (kg N ha^−1^).

## 3. Results

### 3.1. Rainfall and Temperature

**Figure 1:**
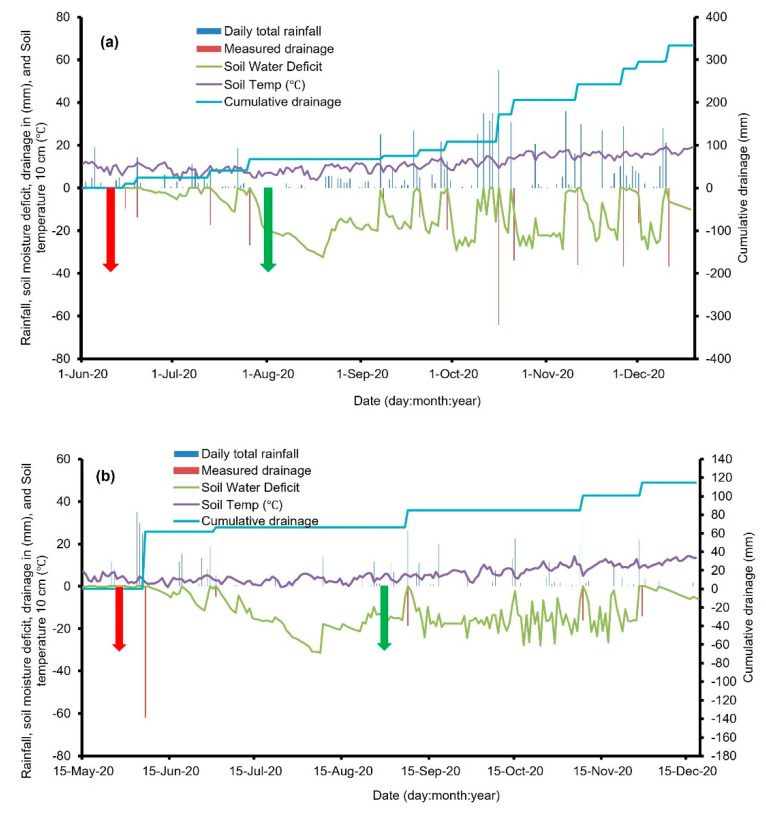
Daily total rainfall, soil water deficit, measured drainage, cumulative drainage, and average soil temperature (10 cm) at the (**a**) Manawatu and (**b**) Canterbury site during the experimental period of May 2020 to December 2020. The red arrow shows the late-autumn treatment application, while the green arrow shows the mid-winter treatment application.

Total rainfall for the Manawatu lysimeters was 805 mm, with 333 mm drainage water collected during the experimental period (9 June 2020 to 15 December 2020) (Figure 1a). Average daily soil temperatures (0–10 cm) for the Manawatu site were below 7 °C for 15 days between July and August and increased to approximately 20 °C in December.

Total rainfall for the Canterbury lysimeters was 314 mm, with 114.9 mm of drainage collected (27 May 2020 to 16 December 2020) (Figure 1b). At the Canterbury site average soil temperatures were below 5 °C from June to August, increasing to about 14 °C in December.

### 3.2. Late-Autumn Treatments Application

#### 3.2.1. Mineral N Leaching Losses

In the Manawatu site, twelve late-autumn leaching events were recorded resulting in cumulative drainage of 333 mm (Figure S2b,c). Maximum leaching occurred at 172.1 mm of cumulative drainage with rates of 21 to 52 kg NO_3_^−^ -N ha^−1^ and from 0.6 to 1.4 kg NH_4_^+^ -N ha^−1^ (Figure S2b,c). The cumulative leached NO_3_^−^ -N and NH_4_^+^ -N in late-autumn urine treatments in the Manawatu lysimeters ranged from 51.8 to 90.7 kg NO_3_^−^ -N ha^−1^ and from 1.4 to 2.1 kg NH_4_^+^ -N ha^−1^ (Table 4). The applied treatments induced significant differences in total mineral N leaching. The DCD, 2LS and GA + LS treatments reduced the total mass of mineral N leaching in the Manawatu site by 8%, 16% and 35%, respectively, compared to the application of the urine-only treatment (Table 4). Whereas, application of PA-MA and LS had no significant effect on total mineral N leaching relative to the application of the urine-only treatment.

In the Canterbury site lysimeters, five late-autumn leaching events were recorded resulting in cumulative drainage of 114.9 mm (Figure S2b,d). Maximum leaching occurred during the first drainage event (between 61.8 mm of cumulative drainage) at rates of 32 to 69 kg NO_3_^−^ -N ha^−1^ and from 36 to 83 kg NH_4_^+^ -N ha^−1^ (Figure S2b,d). The cumulative NO_3_^−^ -N and NH_4_^+^ -N leaching in late-autumn urine treatments ranged from 39.7 to 81.1 kg NO_3_^−^ -N ha^−1^ and from 36.2 to 83.4 kg NH_4_^+^ -N ha^−1^ (Table 4). Reductions from the Canterbury site in total mineral N leaching were 34%, 11%, and 35% for the DCD, 2LS, and GA + LS treatments respectively (*p* < 0.05), relative to the application of the urine-only treatment (Table 1). However, the applications of PA-MA and LS treatments had no significant effect on total mineral N leaching, relative to the application of the urine-only treatment.

#### 3.2.2. Cumulative N Uptake and Cumulative DM Yield

Herbage N uptake and DM yield greatly varied among treatments. Application of DCD, 2LS, and GA + LS treatments to the Manawatu lysimeters induced a nominal but non-significant increase in cumulative N uptake and cumulative DM yield (Table 5) relative to the application of the urine-only treatment.

Similarly, for the Canterbury site there was no significant (*p* > 0.05) increase in cumulative N uptake and cumulative DM yield following application of inhibitors (Table 5) compared to the application of the urine-only treatment.

#### 3.2.3. Soil Mineral N

There were no significant changes in residual soil mineral N between applied inhibitors and the application of the urine-only treatment in either the Manawatu or Canterbury sites (Table S3).

### 3.3. Mid-Winter Treatments Application

#### 3.3.1. Mineral N Leaching Losses

Ten mid-winter leaching events were recorded for the Manawatu site resulting in cumulative drainage of 282.6 mm (Figure S3b,c). Nitrate was the dominate form of N leached. Maximum leaching occurred from the urine-treated lysimeters for a cumulative drainage of 137.3 mm with rates of 46 to58 kg NO_3_^−^ -N ha^−1^ and 0.5 to1 kg NH_4_^+^ -N ha^−1^ (Figure S3b,c). Overall, the GA only and GA + LS treatments significantly (*p* < 0.05) reduced the total amount of mineral N leaching from the Manawatu site by 23% and 20%, respectively, relative to the application of the urine-only treatment (Table 6).

Three leaching events were recorded for the Canterbury site with a cumulative drainage of 48.3 mm (Figure S3b,d). This low drainage resulted in low NO_3_^−^ -N leaching in the different treatments. Maximum leaching from the urine-treated lysimeters was recorded for a cumulative drainage of 34.3 mm with rates of 1.8 to 28.3 kg NO_3_^−^ -N ha^−1^ and 0.1 to 0.6 kg NH_4_^+^ -N ha^−1^ (Figure S3b,d). Overall, lysimeters treated with GA alone showed significant (*p* < 0.05) increases in the mass of N leaching compared to the application of the urine-only treatment. However, there was no significant difference in total mineral N leaching between the urine-only and GA + LS treatments (Table 6).

#### 3.3.2. Cumulative N Uptake and Cumulative DM Yield

Application of GA only and GA + LS treatments to the Manawatu lysimeters showed a significant (*p* < 0.05) increase in cumulative N uptake (22% for GA only and 13% for GA + LS) and cumulative DM yield (18% for GA only and 15% for GA + LS) relative to the application of the urine-only treatment (Table 7). The treatment effect on cumulative N uptake and cumulative DM yield for the Canterbury site was also significant (*p* < 0.05), with corresponding values of 19% and 12% for GA only, and 24% and 19% for GA + LS, respectively (Table 7).

#### 3.3.3. Soil Mineral N

In the Manawatu site, the applied treatments resulted in significant differences in residual soil mineral N in the lysimeters. The soil mineral N was significantly (*p* < 0.05) higher in the GA + LS treatment compared to the application of the urine-only treatments (Table S4). However, there were no significant changes between urine-only and GA treatments.

In the Canterbury site, there were no significant differences in residual mineral N observed between urine-treated lysimeters (Table S4).

## 4. Discussion

### 4.1. Leachate Mineral N

Lysimeter leachate analysis before treatment application (Tables S1 and S2) showed that there was extremely low background mineral N in leachate. Results from our study shows that NO_3_^−^ -N was the major form of N leaching from the Manawatu site for both late-autumn and mid-winter urine applications (Table 4 and Table 6) and this agrees with the general expectation that NO_3_^−^ -N is the predominant form of mineral N in drainage water. However, large quantities of NH_4_^+^ -N leached from the Canterbury lysimeters in late-autumn and this was associated with the first collected drainage (Figure S2d). High NH_4_^+^ -N leachate losses have been previously reported for Canterbury [1]. The authors reported that late-autumn (May) urine application to stony Pallic Orthic Brown soil in Canterbury resulted in NH_4_^+^ -N leaching ranging from 33.0 to 58.7 kg NH_4_^+^ -N ha^−1^, due to urine flowing via macro-pore into the lower gravel layers of the lysimeters. In our study an average of 60.8 kg NH_4_^+^ -N ha^−1^ was leached during the first cumulative drainage of 61.8 mm. The high rainfall event and combination of the free-draining shallow stony soil, limited CEC, and low water holding capacity (40–60 mm) allowed the leaching of NH_4_^+^ -N. In contrast to the Manawatu site, the Orthic Pumice soil can hold between 150–200 mm of water with a higher CEC. In addition to the differences in water holding capacity and CEC between the soils, the stony nature of the Pallic A horizon (0–30 cm, 50–60% stones) allows macro pour flow of urine into the predominantly stone and sand Ap horizon (30–50 cm, 71–75% stones) [26].

The late-autumn application of 2LS, and GA + LS, significantly (*p* < 0.05) reduced the total amount of mineral N leaching from the Manawatu lysimeters relative to the application of the urine-only, while only lysimeters treated with GA + LS showed a significant (*p* < 0.05) reduction in the total mineral N concentration in leachate from the Canterbury lysimeters (Table 4). Application of 2LS proved to be more effective in reducing total mineral N leaching than a single application of LS for the Manawatu site. Therefore, the application of a second dose might have helped to prolong the effectiveness of these compounds in reducing total mineral N losses. However, in the Canterbury lysimeters application of a second dose did not yield reduction in total mineral N leaching. This can an attribute to the fact that a higher proportion of the applied N was leached in the first cumulative drainage event before the second dose application. Further, application of PA-MA and LS treatments resulted in non-significant changes in total mineral N leaching in either Manawatu or Canterbury lysimeters relative to the urine-only treatment. The higher CEC of the Orthic Pumice soil might support the adsorption of inhibitors to soil organic matter [27]. On the other hand the low CEC and low water holding capacity of the Pallic Orthic Brown soil might have exacerbated the possibilities of leaching of these inhibitors during drainage [28]. These factors might have contributed to the reduction of the inhibitor’s effectiveness.

The combination of GA + LS treatment reduced total mineral N leaching in both the Manawatu and Canterbury sites. In this study, GA was applied to improve N uptake and plant growth as a complimentary mechanism to the effect of LS. First herbage cut N uptake data from both sites suggests that this combination might have reduced total N losses through increasing N uptake when compared to the other treatments (Tables S5 and S6). This increase in herbage N uptake may have resulted in less soil mineral N available to leaching during drainage events. However, future studies are needed to provide clear evidence on the mode of action of this treatment. A similar study [18], also found that late-autumn GA + LS application significantly reduced NO_3_^−^ -N leaching in the Orthic Pumice soil and Pallic Orthic Brown soil by 15% and 22%, respectively.

Mid-winter application of GA alone and GA + LS significantly (*p* < 0.05) reduced total N leaching loss from the Manawatu site (Italian ryegrass). However, the same result was not observed for the Canterbury site (perennial ryegrass/clover mixture) where GA alone increased total mineral N leaching and GA + LS had no significant effect when compared to urine-only (Table 6). The increase in N leaching in the Canterbury site associated with GA alone might be attributed to the interaction between the GA and white clover. Several studies have provided evidence that the application of GA increases nodule formation in legumes [29,30] and high nodulation in legumes can increase biological nitrogen fixation (BNF). Application of GA_3_ (10^−5^ M) as foliar spray to *Rhizobium* inoculated chickpea plants and significantly increased nodules per plant by 55% relative to the control [30]. Increased BNF by *Rhizobium* bacteria associated with clover nodules might have reduced the utilisation of urine applied N, thus making it susceptible to leaching. Further, the increase in nitrogen fixation might lead to an increase in the total N input and eventually increasing the NO_3_^−^ -N leaching potential. Reduced N leaching by GA + LS was a combination effect of LS and the complementary effect of GA. Evidence for this theory is the significantly (*p* < 0.05) higher cumulative N uptake due to the GA + LS treatment in this study when compared to GA alone (Table 6).

### 4.2. Pasture N Uptake and DM Yield

The application of late-autumn inhibitors did not lead to any significant increase in cumulative herbage N uptake and cumulative DM yield for either of the lysimeter sites (Table 5) when compared to the urine-only lysimeters. The non-significant increase in cumulative N uptake and cumulative DM yield associated with the applied treatments was influenced by the form of N present in both soils. The complete nitrification in soil occurs within 2–4 weeks when conditions are favourable [31]. Converting NH_4_^+^ -N to NO_3_^−^ -N which is rapidly available to plants due to its high mobility in soil. However, plants must reduce nitrate to its amine form prior to the biosynthesis of proteins, this requires more energy than the utilisation of either urea or NH_4_^+^ ions [32]. Previous studies have also reported on inhibitors reducing NO_3_^−^ -N leaching; however, they did not show a significant effect on cumulative N uptake and pasture DM [32,33], due to suppression of soil NO_3_^−^ -N levels.

Although, the applied treatments did not result in an overall significant cumulative N uptake and DM yield between treatments, significant treatment effects were observed during the first harvest dates. For example, late-autumn application of DCD, 2LS, and GA + LS significantly (*p* < 0.05) increased herbage N uptake and DM yield for the first harvest in the Manawatu site compared to the urine-only treatment (Table S5). For the Canterbury lysimeters this increase was non-significant. The higher herbage N uptake in the first cut demonstrated that these treatments were effective in delaying the oxidation of NH_4_^+^ -N during the period of rapid nitrification; however, their short effectiveness might be due to rapid degradation in the soil [34,35].

Mid-winter application of GA alone and GA + LS significantly (*p* < 0.05) increased both cumulative N uptake and DM yield in both the Manawatu and Canterbury sites (Table 7) relative to the urine-only treated lysimeters. The treatments effect in the Manawatu lysimeters was due to the long period between the urine application and the first leaching event (Figure 1a). The longer period allowed high utilisation of applied N by lysimeter pasture thus, giving significant differences between the applied treatments. The effectiveness of these treatments in the Canterbury site might have been accelerated by the low total mass of N leached from the Canterbury lysimeters. As a result, a higher proportion of N was available for plant uptake.

### 4.3. Soil Mineral N and N Recovered in the System

Soil mineral N results analysed at the end of this current study showed that there was no significant difference between urinetreated and untreated lysimeters at both experimental sites with either late-autumn or mid-winter treatment application. This implies that all applied urine N was either utilised through pasture N uptake or lost through leaching or any other possible pathways such as immobilisation or emissions. In this study the recovered N calculations showed that an average of 0.01%, 13.09%, and 31.51% of the applied urine N in the Manawatu site (late-autumn treatments) was recovered through soil residual N, leached N, and herbage N uptake, respectively (Table 8). While in the Canterbury site, soil residual N, leached N, and herbage N uptake was 1.66%, 19.07%, and 35.29%, respectively of the applied urine N. In the Manawatu and Canterbury sites, the unaccounted N was 55.38% and 43.98%, respectively of the applied urine N. Further, mid-winter treatments in the Manawatu lysimeters showed that an average of 0.41%, 19.00%, and 29.87% of applied urine N was recovered through soil residual N, leached N, and herbage N uptake, respectively, while unaccounted N was 50.73%. Similarly, in the Canterbury site, average N recovered in soil, leaching, and herbage was 4.99%, 7.57%, and 49.44%, respectively. Unaccounted N corresponded to 49.44% in the Canterbury site (Table 8). The unaccounted N is mainly N lost through immobilisation in the soil microbial biomass and organic matter or through emissions. In this current study, unaccounted N was nearly 50% and this percentage has been reported in previous studies. In literature, an average of 26%, 13%, and 2% of applied urinary N has been reported to be lost through immobilisation, NH_3_ volatilisation, and N_2_O emissions [36]. In a field lysimeter study reported by Zaman and Blennerhassett [32], the unaccounted N was 60.29% and 56.69% in autumn and spring, respectively. The values of urine applied N recovered through herbage N uptake in this study agree with other studies who observed similar trends [37,38]. For example, Ball et al. [37] reported that urine applied at 300 kg N ha^−1^, the N recovered through plant N uptake was 37% of the applied urine-N. Overall, a higher percentage of unaccounted N was in the Manawatu lysimeters. The differences in soil properties between the two sites might have played a major role in influencing this trend. In this current study, the wet conditions (Figure 1a,b) between June to August in both experimental sites, might have resulted in an increase in the population of denitrifying microorganisms. Denitrifying microbes might have released N from soil as N_2_O and N_2_ gases, leading to poor soil N utilisation by the pasture. Emissions can reach up to 28% of applied N due to the wet conditions which prevail between May and early July [39]. However, emissions were not measured in this current study, and this is an area for future work.

### 4.4. Importance of These Findings

Overall, our field study results showed that the two soils in the different locations present different potentials to NO_3_^−^ -N leaching. According to the N recovery results, leached N in late-autumn applied treatments accounted for 19.07% in the Canterbury site compared to 13.09% recorded in the Manawatu site. These findings provide clear evidence that the Canterbury site (Pallic Orthic Brown soil) poses a greater threat to N loss through leaching. While the Orthic Pumice soil in the Manawatu site showed a lower herbage N uptake and higher proportion of unaccounted N. This shows a higher potential for N loss through emissions and immobilization of N. This information is important for the proper implementation of management practices. In terms of treatment effects, our results showed that the application of 2LS, and GA + LS during May to December was effective in reducing NO_3_^−^ -N leaching in two different locations with different soils and under different management conditions. These findings demonstrate the potential of these treatments in reducing NO_3_^−^ -N leaching within the different regions of New Zealand. Since this was the first published study conducted using these inhibitors (LS and PA-MA) it was imperative to provide information on the effectiveness of these treatments under different environment, climatic conditions, soils, and grasses. The inclusion of GA also provided critical insight on the possible manipulation of plant growth as a strategy to reduce NO_3_^−^ -N leaching in urine patches.

Further research is to be conducted on the direct effect of these applied inhibitors on *amoA* gene abundance in the soil and possibly N_2_O emissions.

## 5. Conclusions

This study demonstrated that the split application of calcium lignosulphonate significantly (*p* < 0.05) reduced total mineral N leaching only in the Manawatu site, whereas gibberellic acid plus calcium lignosulphonate treatment significantly (*p* < 0.05) reduced mineral N leaching in both Manawatu and Canterbury site lysimeters. These treatments provided valuable evidence on potential amendments that can be applied to urine patches to reduce mineral N leaching losses. The study showed that a split application of calcium lignosulphonate reduced N leaching by means of increasing the calcium lignosulphonate reactive period in the soil while the reduction associated with gibberellic acid plus calcium lignosulphonate treatment was due to a combination of calcium lignosulphonate and gibberellic acid effect. The timing of treatment is important, with the late-autumn application showing higher efficacy in reducing N leaching from both soils than the mid-winter application. Our results have demonstrated that for farmers to achieve the greatest reduction in N leaching during the period of high N losses and drainage, application of an inhibitor is necessary during the late-autumn period. Our findings can potentially guide farm management practices with respect to the optimal timing of nitrification inhibitor application to grazed pastoral systems.

## Figures and Tables

**Table 1:** Selected soil basic properties analysed prior to treatment application.

Parameter	Orthic Pumice Soil (Manawatu Site)	Pallic Orthic Brown Soil (Canterbury Site)
pH	5.85	5.12
% N	0.20	0.36
% C	3.68	4.20
% Al	0.85	0.30
% Fe	0.30	0.38
Exchangeable Cations (meq 100 mL^−1^)		
Ca	28	1
K	1.71	0.22
Mg	0.32	0.90
Na	0.12	0.11
^1^ CEC	22.1	9.4
^2^ WHC (%)	80.6	45.9

^1^ CEC = Cation Exchange Capacity; ^2^ WHC = Water Holding Capacity.

**Table 2:** Description of late-autumn treatments applied in the Manawatu lysimeters on 9 June 2020 and in the Canterbury lysimeters on 27 May 2020.

Late-Autumn Treatments	Urine N Rate (kg N ha^−1^)	Replicates
Control (water)	Nil	4
Urine-only	600	4
Urine + DCD at 10 kg ha^−1^	600	4
Urine + PA-MA at 10 kg ha^−1^	600	4
Urine + LS at 120 kg ha^−1^	600	4
Urine + split-application of LS (2LS) at same rate initial and after a month of first application	600	4
Urine + GA (ProGibb SG at 80 g ha^−1^) + LS at 120 kg ha^−1^	600	4

**Table 3:** Description of mid-winter treatments applied in the Manawatu lysimeters on 29 July 2020 and in the Canterbury lysimeters on 26 August 2020.

Late-Autumn Treatments	Urine N Rate (kg N ha^−1^)	Replicates
Control (water)	Nil	4
Urine-only	600	4
Urine + GA (ProGibb SG at 80 g ha^−1^)	600	4
Urine + GA (ProGibb SG at 80 g ha^−1^) + LS at 120 kg ha^−1^	600	4

**Table 4:** Cumulative NO_3_^−^ -N leaching, cumulative NH_4_^+^ -N leaching, and total mineral N following late-autumn treatment application in the Manawatu site for the period 9 June 2020 to 15 December 2020 and Canterbury site for the period 27 May to 16 December 2020.

	Manawatu Site			Canterbury Site		
Treatments	Cumulative Nitrate Leaching kg NO_3_^−^ -N ha^−1^	Cumulative Ammonia Leaching kg NH_4_^+^ -N ha^−1^	Total Mineral N Leaching (kg N ha^−1^)	Cumulative Nitrate Leaching kg NO_3_^−^ -N ha^-^	Cumulative Ammonia Leaching kg NH_4_^+^ -N ha^−1^	Total Mineral N Leaching (kg N ha^−1^)
Control	6.9 ± 0.27 e	1.8 ± 0.12 ab	8.7 ± 0.51 d	10.5 ± 1.15 d	3.3 ± 0.16 d	13.7 ± 0.68 d
Urine-only	84.3 ± 2.75 ab	1.4 ± 0.09 b	85.7 ± 2.96 a	62.9 ± 4.69 b	83.4 ± 2.68 a	146.3 ± 5.65 ab
Urine + DCD	76.6 ± 2.60 bc	1.9 ± 0.11 ab	78.5 ± 4.62 ab	39.7 ± 3.35 c	57.7 ± 1,27 bc	97.3 ± 9.75 bc
Urine + PA-MA	90.7 ± 1.97 a	1.4 ± 0.90 b	92.1 ± 3.05 a	81.1 ± 0.70 a	68.4 ± 1.50 ab	149.5 ± 8.17 a
Urine + LS	87.4 ± 3.05 ab	1.7 ± 0.25 ab	89.1 ± 2.16 a	76.5 ± 1.84 a	75.0 ± 2.71 ab	151.5 ± 5.75 a
Urine + 2LS	70.4 ± 1.53 c	1.7 ± 0.23 ab	72.1 ± 2.69 b	58.4 ± 3.02 b	71.4 ± 1.70 ab	130.1 ± 8.29 abc
Urine + GA + LS	51.8 ± 3.11 d	2.1 ± 0.07 a	53.9 ± 3.62 c	50.4 ± 3.29 bc	44.0 ± 2.31 c	94.5 ± 1.06 c

Note: Numbers after ± represent standard error of mean. Different small letters in each column of each soil indicate a significant difference at *p* < 0.05.

**Table 5:** Cumulative N uptake (kg N ha^−1^) and cumulative DM yield (kg DM ha^−1^) following late-autumn treatment application in the Manawatu site for the period 9 June 2020 to 15 December 2020 and Canterbury site for the period 27 May to 16 December 2020.

	Manawatu Site		Canterbury Site	
Treatments	Cumulative N Uptake (kg N ha^−1^)	Cumulative DM Yield (kg DM ha^−1^)	Cumulative N Uptake (kg N ha^−1^)	Cumulative DM Yield (kg DM ha^−1^)
Control	48.2 ± 1.17 d	2783 ± 176 b	93.2 ± 9.45 b	4421 ± 300 b
Urine-only	232.5 ± 2.56 ab	9568 ± 156 a	280.9 ± 13.9 a	10,106 ± 421 a
Urine + DCD	254.8 ± 15.70 a	10,276 ± 669 a	327.3 ± 21.50 a	10,971 ± 743 a
Urine + PA-MA	204.0 ± 7.29 c	9941 ± 596 a	286.4 ± 11.90 a	10,223 ± 343 a
Urine + LS	213.2 ± 11.40 bc	9474 ± 562 a	308.1 ± 20.10 a	10,596 ± 842 a
Urine + 2LS	258.1 ± 16.00 a	10,301 ± 719 a	312.5 ± 21.40 a	10,892 ± 1080 a
Urine + GA + LS	261.1 ± 7.02 a	9583 ± 885 a	314.4 ± 19.20 a	11,286 ± 606 a

Note: Numbers after ± represent standard error of mean. Different small letters in each column of each soil indicate a significant difference at *p* < 0.05.

**Table 6:** Cumulative NO_3_^−^ -N leaching, cumulative NH_4_^+^ -N leaching, and total mineral N following mid-winter treatment application in the Manawatu site for the period 29 July 2020 to 15 December 2020 and Canterbury site for the period 26 August 2020 to 16 December 2020.

	Manawatu Site			Canterbury Site		
Treatments	Cumulative Nitrate Leached kg NO_3_^−^ -N ha^−1^	Cumulative Ammonia Leaching kg NH_4_^+^ -N ha^−1^	Total Mineral N Leaching (kg N ha^−1^)	Cumulative Nitrate Leaching kg NO_3_^−^ -N ha^-^	Cumulative Ammonia Leaching kg NH_4_^+^ -N ha^−1^	Total Mineral N Leaching (kg N ha^−1^)
Control	2.5 ± 0.16 c	1.4 ± 0.18 a	3.9 ± 0.02 c	8.3 ± 0.47 c	0.2 ± 0.07 bc	8.5 ± 0.41 c
Urine-only	136.7 ± 3.04 a	0.9 ± 0.07 a	137.8 ± 2.97 a	53.1 ± 0.83 b	0.7 ± 0.04 a	53.8 ± 0.82 b
Urine + GA	104.8 ± 2.66 b	0.9 ± 0.19 a	105.6 ± 2.61 b	57.1 ± 0.94 a	0.2 ± 0.01 c	57.3 ± 0.93 a
Urine + GA + LS	109.0 ± 4.38 b	1.1 ± 0.32 a	110.1 ± 2.97 b	50.5 ± 0.86 b	0.4 ± a0.06 ab	50.9 ± 0.82 b

Note: Numbers after ± represent standard error of mean. Different small letters in each column of each soil indicate a significant difference at *p* < 0.05.

**Table 7:** Cumulative N uptake (kg N ha^−1^) and cumulative DM yield (kg DM ha^−1^) following mid-winter treatment application in the Manawatu site for the period 29 July 2020 to 15 December 2020 and Canterbury site for the period 26 August 2020 to 16 December 2020.

	Manawatu Site		Canterbury Site	
Treatments	Cumulative N Uptake (kg N ha^−1^)	Cumulative DM Yield (kg DM ha^−1^)	Cumulative N Uptake (kg N ha^−1^)	Cumulative DM Yield (kg DM ha^−1^)
Control	45.5 ± 0.88 c	2692 ± 166 c	81.8 ± 8.76 d	3992 ± 425 d
Urine-only	201.0 ± 5.23 b	8061 ± 380 b	271.5 ± 18.30 c	9405 ± 719 c
Urine + GA	245.8 ± 9.19 a	9519 ± 141 a	321.9 ± 11.00 b	10,506 ± 328 b
Urine + GA + LS	227.3 ± 15.60 a	9270 ± 526 a	336.0 ± 17.70 a	11,148 ± 755 a

Note: Numbers after ± represent standard error of mean. Different small letters in each column of each soil indicate a significant difference at *p* < 0.05.

**Table 8:** Percentage (%) of applied N recovered in soil, herbage, leachate, and unaccounted N in the late-autumn and mid-winter treatments urine application.

Treatments	Residual Soil Mineral N	Herbage N Uptake	Leached N	Unaccounted N
**Late-autumn application**				
Manawatu site	0.01	31.51	13.09	55.38
Canterbury site	1.66	35.29	19.07	43.98
**Mid-winter application**				
Manawatu site	0.41	29.87	19.00	50.73
Canterbury site	4.99	38.00	7.57	49.44

## Data Availability

The analysed data during this current study is not publicly available because it is part of the first author’s graduation thesis, but can be available from the corresponding author upon reasonable request.

## Supplementary Materials

The following supporting information can be downloaded at: https://www.mdpi.com/article/10.3390/plants11182430/s1, Figure S1: Showing the location of the lysimeter sites in the South and North Island of New Zealand; Figure S2: Leached NO_3_^−^ -N and NH_4_^+^ -N from the Manawatu and Canterbury sites as a function of cumulative drainage following late-autumn treatments application; Figure S3: Leached NO_3_^−^ -N and NH_4_^+^ -N from the Manawatu and Canterbury sites as a function of cumulative drainage following mid-winter treatments application; Table S1: NO_3_^−^ -N in leachate in the late-autumn treatment application in both sites before treatment application; Table S2: NO_3_^−^ -N in leachate in the mid-winter treatment application in both sites before treatment application; Table S3: Soil NO_3_^−^ -N, NH_4_^+^ -N, and soil total mineral N analysed at the end of the experiment following late-autumn treatment application in both experimental sites; Table S4: Soil NO_3_^−^ -N, NH_4_^+^ -N, and soil total mineral N analysed at the end of the experiment following mid-winter treatment application in both experimental sites; Table S5: Herbage N uptake and herbage DM yield following late-autumn treatment application to the Manawatu and Canterbury sites; Table S6: Herbage N uptake and herbage DM yield following mid-winter treatment application to the Manawatu and Canterbury sites.
