# Supplementary material for: Nitrate Leaching Mitigation Options in Two Dairy Pastoral Soils and Climatic Conditions in New Zealand
Source: Plants (Basel). 2022 Sep 17;11(18):2430. doi: 10.3390/plants11182430 (PMC9502902; doi:10.3390/plants11182430)
Supplement: Supplementary file 1 [file plants-11-02430-s001.zip › plants-1892969-supplementary.pdf]

Nitrate leaching mitigation options in two dairy pastoral soils under different climatic conditions in New Zealand

Dumsane Themba Matse, Paramsothy Jeyakumar*, Peter Bishop and Christopher W N Anderson

Environmental Science Group, School of Agriculture and Environment, Massey University, Private Bag 11 222, Palmerston North 4442, New Zealand

*Corresponding author: Paramsothy Jeyakumar

Email: P.Jeyakumar@massey.ac.nz; Tel: +6421814545

Supplementary material (3 Figures and 6 Tables)

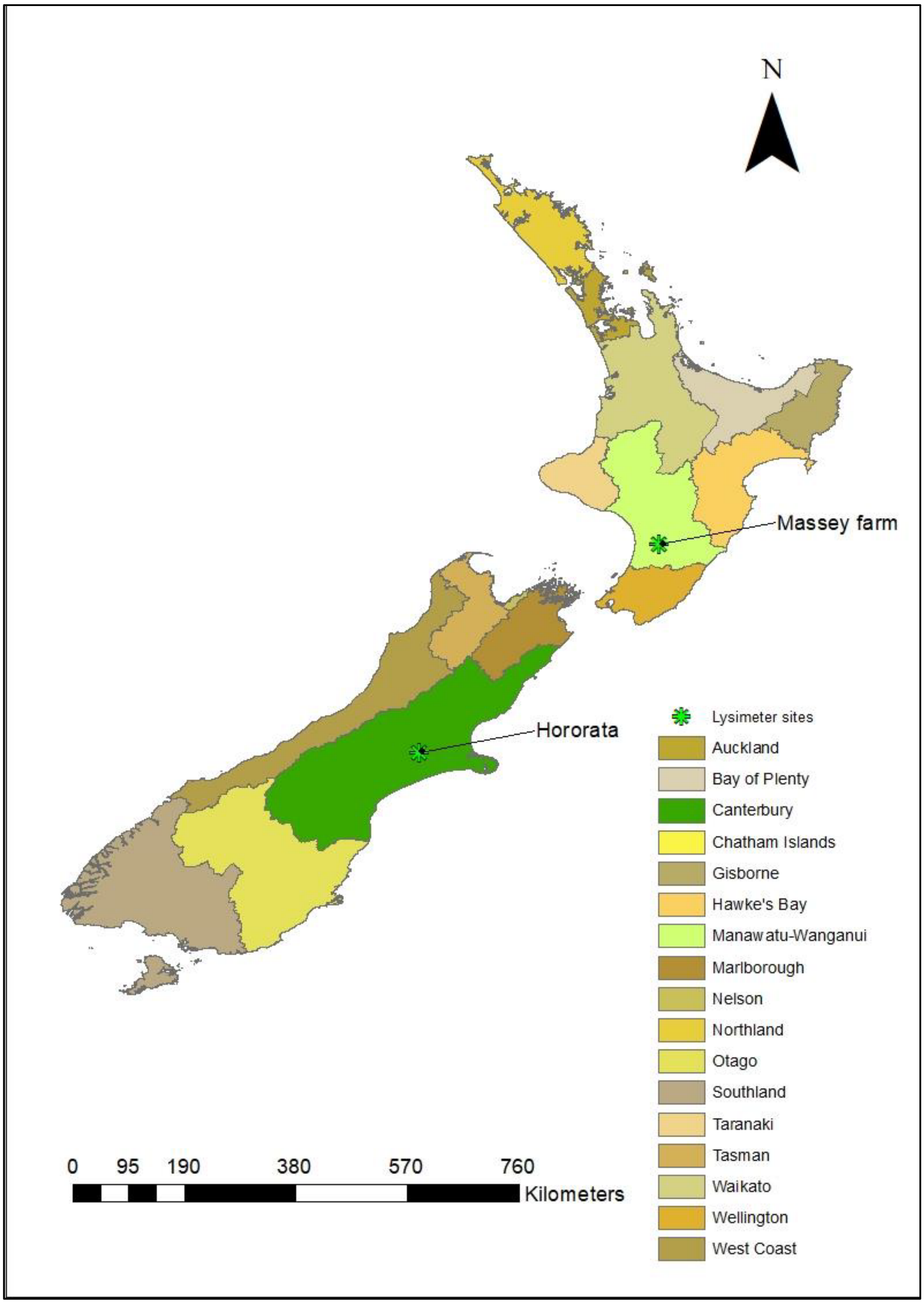

Figure S1. Green insert stars show the location of the Lysimeter sites in the South and North Island of New Zealand.

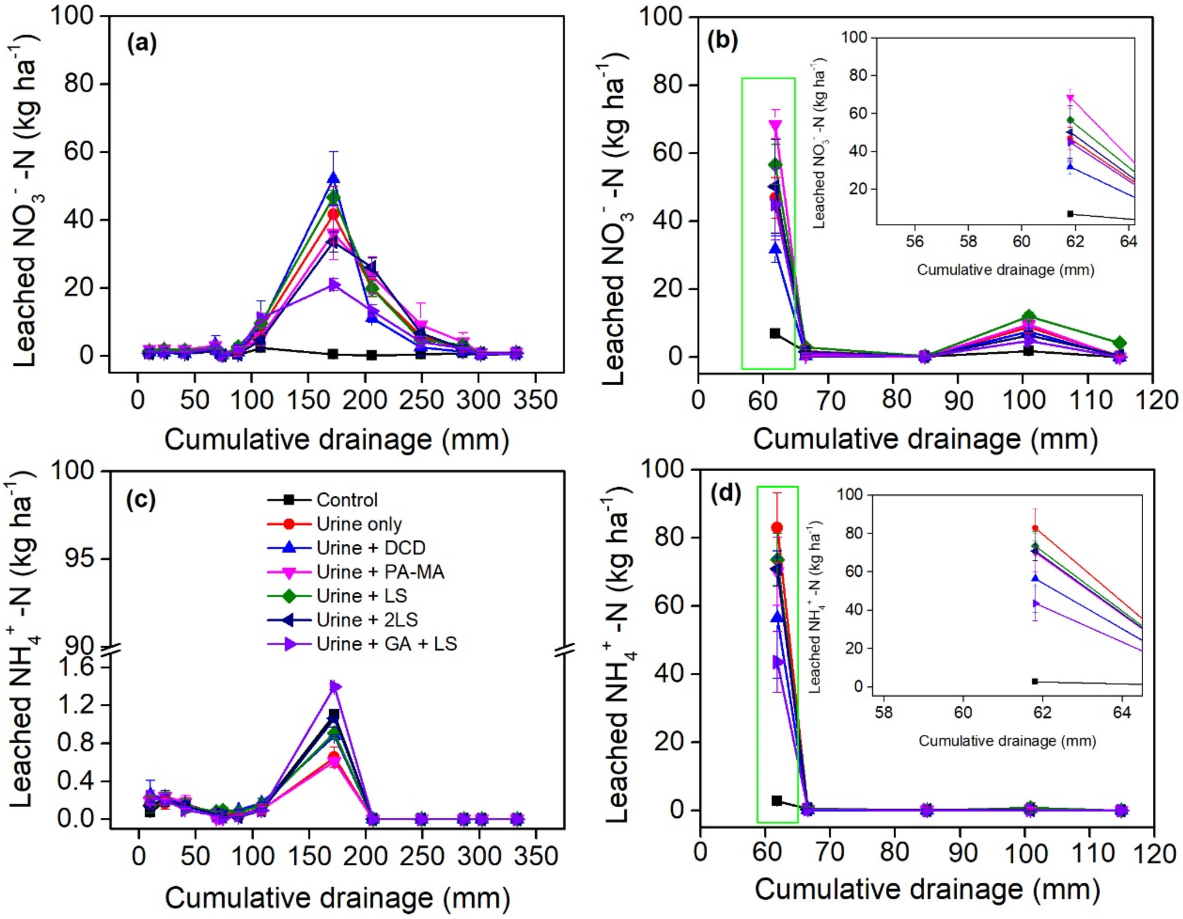

Figure S2. Leached NO_3^- -N from the Manawatu (**a**) and Canterbury site (**b**), and leached NH_4^+ -N from the Manawatu (**c**) and Canterbury lysimeters (**d**) as a function of cumulative drainage following late-autumn urine and treatments application to lysimeters. Error bars represent standard deviation of mean (*n* = 4). Data points for the Canterbury lysimeters start at 61.8 cumulative drainage and correspond to both the maximum and first collected drainage for the late-autumn treatment application.

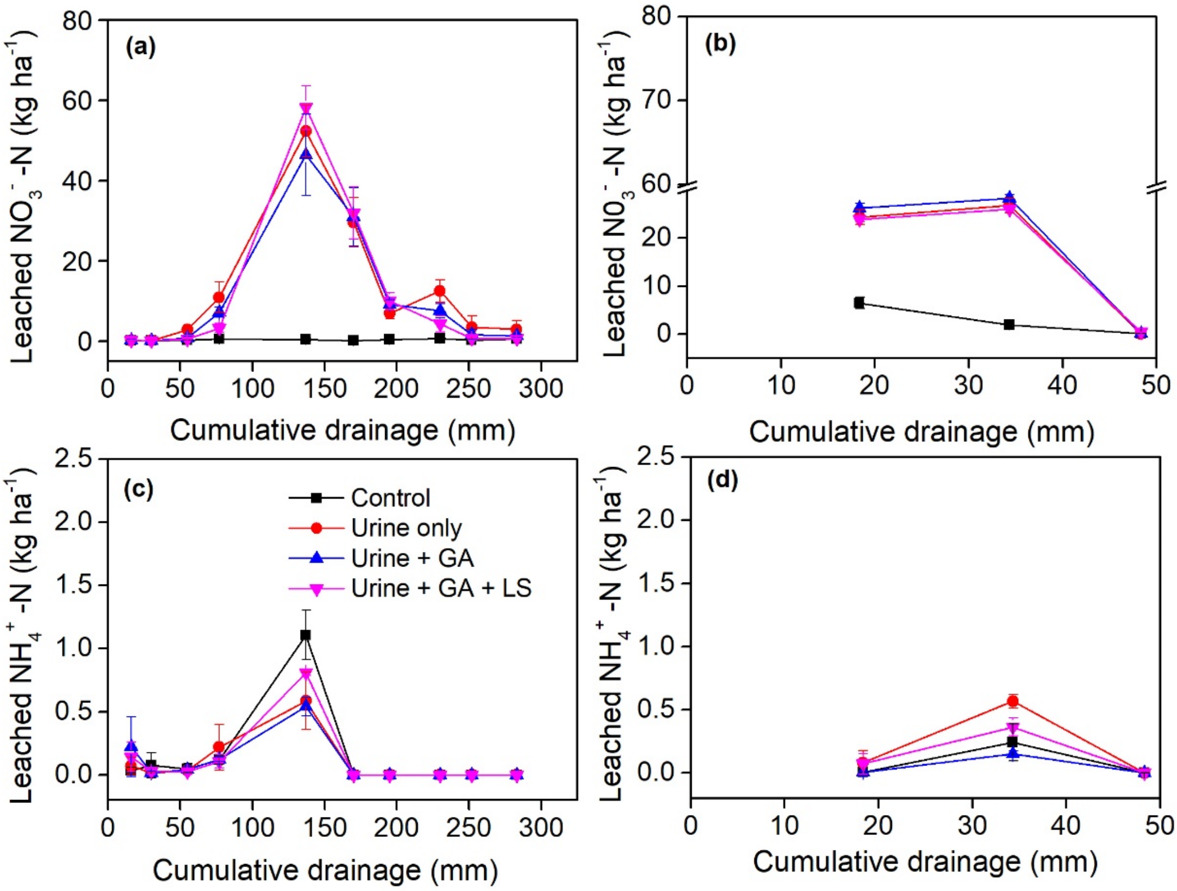

Figure S3. Leached NO_3^- -N from the Manawatu (**a**) and Canterbury lysimeters (**b**), and leached NH_4^+ -N from the Manawatu (**c**) and Canterbury lysimeters (**d**) as a function of cumulative drainage following mid-winter urine and treatments application to lysimeters. NH_4^+ -N concentration after 150 mm cumulative drainage from the Manawatu lysimeters was below detectable levels. Vertical error bars represent standard deviation of means (*n* = 4). Data points for the Canterbury lysimeters start at 18.4 mm cumulative drainage and correspond to the first collected drainage for the mid-winter treatment application.

Table S1. NO_3^- -N in leachate in the late-autumn treatments application in the Manawatu and Canterbury lysimeters before treatment application.

Treatments	Manawatu lysimeters	
	**05/06/2020**	**09/06/202**
	**Leachate kg NO_3^- -N ha^{-1}**	**Leachate kg NO_3^- -N ha^{-1}**
Control	1.0±0.23a	1.4±0.75a
Urine only	1.4±0.26a	1.5±0.39a
Urine + DCD	1.1±0.37a	1.5±0.09a
Urine + PA-MA	1.6±0.90a	1.1±0.44a
Urine + LS	1.3±0.29a	1.4±0.73a
Urine + 2LS	2.0±0.69a	1.2±0.69a
Urine + GA + LS	0.9±0.27a	0.9±0.19a
**Treatments**	**Canterbury lysimeters**	
	**13/05/2020**	**20/05/2020**
	**Leachate kg NO_3^- -N ha^{-1}**	**Leachate kg NO_3^- -N ha^{-1}**
Control	8.5±2.37a	1.0±0.23
Urine only	9.7±1.46a	1.4±0.26
Urine + DCD	9.4±1.38a	1.6±1.33
Urine + PA-MA	11.3±1.91a	1.6±0.90
Urine + LS	9.4±1.73a	1.5±0.61
Urine + 2LS	9.6±1.73a	1.9±0.69
Urine + GA + LS	8.7±1.15a	1.0±0.74

Values after ±represent standard deviation. Different small letters in each soil column indicate significant difference at $P < 0.05$. NH_4^+ -N was below detectable concentrations.

Table S2. NO_3^- -N in leachate in the mid-winter treatments application in the Manawatu and Canterbury lysimeters before treatments application.

**Treatments**	**Manawatu lysimeters**	
	**05/06/2020**	**09/06/202**
	**Leachate kg NO_3^- -N ha^{-1}**	**Leachate kg NO_3^- -N ha^{-1}**
Control	1.2±0.21a	1.0±0.30a
Urine only	0.9±0.52a	1.0±0.20a
Urine + GA	1.4±0.93a	1.2±0.18a
Urine + GA + LS	1.3±0.90a	1.0±0.58a
**Treatments**	**Canterbury lysimeters**	
	**13/05/2020**	**20/05/2020**
	**Leachate kg NO_3^- -N ha^{-1}**	**Leachate kg NO_3^- -N ha^{-1}**
Control	10.1±1.04a	0.9±0.60a
Urine only	11.2±1.55a	1.0±0.42a
Urine + GA	10.9±1.06a	0.9±0.50a
Urine + GA + LS	9.7±0.35a	1.3±0.90a

Values after ±represent standard deviation. Different small letters in each soil column indicate significant difference at $P < 0.05$. NH_4^+ -N was below detectable concentrations.

Table S3. Soil NO_3^--N, NH_4^+-N, and soil total mineral N analysed at the end of the experiment following late-autumn treatment application in the Manawatu and Canterbury lysimeters.

	**Manawatu lysimeters**		
Treatments	NO_3^--N (kg ha^{-1})	NH_4^+-N (kg ha^{-1})	Total mineral N (kg N ha^{-1})
Control	15.8±1.6a	0.13±0.02b	16.0±1.6a
Urine only	16.2±2.7a	0.13±0.03ab	16.3±2.7a
Urine + DCD	18.6±3.9a	0.18±0.01a	18.7±3.9a
Urine + PA-MA	16.9±3.3a	0.14±0.02ab	17.1±3.3a
Urine + LS	14.3±2.0a	0.15±0.02ab	14.4±2.0a
Urine + 2LS	12.7±1.0a	0.18±0.02a	12.9±1.0a
Urine + GA + LS	16.6±3.0a	0.13±0.04ab	16.7±3.0a
	**Canterbury lysimeters**		
Treatments	NO_3^--N (kg ha^{-1})	NH_4^+-N (kg ha^{-1})	Total Mineral N (kg N ha^{-1})
Control	17.4±3.7b	11.4±1.7a	28.8±4.3b
Urine only	31.7±5.8ab	3.8±0.7bc	35.5±6.4ab
Urine + DCD	29.1±3.7ab	3.1±0.5c	32.2±2.5b
Urine + PA-MA	35.3±4.8a	5.4±1.2b	40.7±1.9ab
Urine + LS	31.9±5.4ab	9.6±0.4c	34.6±6.1b
Urine + 2LS	40.6±10.4a	9.6±1.0a	50.2±10.5a
Urine + GA + LS	36.3±8.9a	2.8±0.9c	39.2±8.7ab

Numbers after ± represent standard deviation. Different small letters in each column of each soil indicate significant difference at $P < 0.05$.

Table S4. Soil NO_3^- -N, NH_4^+ -N, and soil total mineral N analysed at the end of the experiment following mid-winter treatment application in the Manawatu and Canterbury lysimeters.

	**Manawatu lysimeters**		
Treatments	NO_3^- -N (kg ha^{-1})	NH_4^+ -N (kg ha^{-1})	Total mineral N (kg N ha^{-1})
Control	15.8±1.6b	1.1±0.1a	16.9±1.5b
Urine only	14.0±2.9b	0.7±0.1b	14.8±2.9b
Urine + GA	18.2±2.6ab	1.1±0.2a	19.3±2.5ab
Urine + GA + LS	22.7±4.0a	1.3±0.1a	24.0±3.9a
	**Canterbury lysimeters**		
Treatments	NO_3^- -N (kg ha^{-1})	NH_4^+ -N (kg ha^{-1})	Total Mineral N (kg N ha^{-1})
Control	17.4±3.1b	11.4±1.7a	28.8±4.3a
Urine only	58.0±4.5a	4.9±1.2b	62.9±5.6b
Urine + GA	49.1±8.0a	4.3±1.2b	53.4±9.0b
Urine + GA + LS	54.1±6.2a	5.9±0.8b	60.0±7.0b

Numbers after ± represent standard deviation. Different small letters in each column of each soil indicate significant difference at $P < 0.05$.

Table S5. Herbage N uptake (kg N/ha) and herbage DM yield (kg DM/ha), following late-autumn urine and treatment application to the Manawatu and Canterbury site

**Manawatu site**										
Treatments	11/07/20		11/09/20		10/10/20		11/11/20		11/12/20	
	kg N/ha	kg DM/ha	kg N/ha	kg DM/ha	kg N/ha	kg DM/ha	kg N/ha	kg DM/ha	kg N/ha	kg DM/ha
Control	2.7d	91e	1.8d	121b	11.6c	538d	11.0a	772b	21.4a	1262b
Urine only	11.6c	266d	95.2b	3062a	66.8b	2663c	31.7ab	1968a	24.6a	1520a
Urine + DCD	16.8b	373b	95.3b	3303a	79.5ab	2815bc	35.7a	2088a	27.4a	1697a
Urine + PA-MA	11.7c	274dc	62.9c	3196a	76.2ab	3067ab	28.9b	1888a	24.5a	1515a
Urine + LS	13.9c	320c	67.6c	2857a	72.8ab	2614c	32.2ab	1975a	26.7a	1708a
Urine + 2LS	16.9b	385b	101.8b	3190a	85.4a	3276a	28.7b	1860a	25.3a	1590a
Urine + GA + LS	21.3a	505a	114.9a	2993a	68.5b	2527c	30.8ab	1897a	25.6a	1661a
**Canterbury site**										
			27/08/20		17/10/20		19/11/20		16/12/20	
Treatments			kg N/ha	kg DM/ha	kg N/ha	kg DM/ha	kg N/ha	kg DM/ha	kg N/ha	kg DM/ha
Control			11.4c	454d	27.1c	1002b	36.8b	2212b	18.0d	777b
Urine only			76.8a	1964ab	74.6b	3122a	91.9a	3798a	37.6c	1221a
Urine + DCD			80.80a	2125a	93.7a	3098a	103.4a	4114a	46.9b	1634a
Urine + PA-MA			61.8b	1693abc	88.8a	2934a	94.4a	4275a	41.6bc	1321a
Urine + LS			54.8b	1450bc	95.9a	3013a	102.4a	4483a	55.0a	1650a
Urine + 2LS			56.7b	1276c	96.4a	3731a	116.6a	4445a	42.2bc	1440a
Urine + GA + LS			80.7a	2144a	89.1a	3161a	101.8a	4541a	42.8bc	1441a

Values in each column, followed by different small letters within a column for each soil, are significantly different at $P < 0.05$.

Table S6. Herbage N uptake (kg N/ha) and herbage DM yield (kg DM/ha) following mid-winter urine and treatment application to the Manawatu and Canterbury site.

**Manawatu site**								
	11/09/20		10/10/20		11/11/20		10/12/20	
Treatments	kg N/ha	kg DM/ha	kg N/ha	kg DM/ha	kg N/ha	kg DM/ha	kg N/ha	kg DM/ha
Control	1.8c	121c	11.6c	538b	11.0b	772c	21.4a	1262b
Urine only	33.6b	867b	94.5a	2986a	47.4a	2682b	25.6a	1527a
Urine + GA	57.2a	1554a	102.5a	3037a	57.8a	3157a	28.4a	1771a
Urine + GA + LS	52.6a	1428a	96.8a	3031a	52.5a	3107a	25.3a	1704a
**Canterbury site**								
			17/10/20		16/11/20		16/12/20	
Treatments			kg N/ha	kg DM/ha	kg N/ha	kg DM/ha	kg N/ha	kg DM/ha
Control			27.1c	1002b	36.8c	2212c	18.0c	777c
Urine only			125.6b	3675a	87.6b	3968b	58.3b	1762b
Urine + GA			119.7b	3667a	130.6a	4802a	71.5a	2040a
Urine + GA + LS			139.3a	4032a	124.3a	5123a	72.4a	1993a

Values in each column, followed by different small letters within a column for each soil, are significantly different at $P < 0.05$.
